# Impaired cell viability and mitochondrial respiration by disperse textile dyes

**DOI:** 10.3389/falgy.2025.1547225

**Published:** 2025-04-24

**Authors:** Lizette M. Cortes, Nelson R. Vinueza

**Affiliations:** ^1^ Department of Molecular Biomedical Sciences, North Carolina State University, Raleigh, NC, United States; ^2^ Department of Textile Engineering, North Carolina State University, Raleigh, NC, United States; ^3^ Department of Chemistry, North Carolina State University, Raleigh, NC, United States

**Keywords:** textile dyes, cell viability, mitochondrial respiration, disperse dyes, D. Blue 124, D. Blue 1, D. Brown 1

## Abstract

In recent years, the use of synthetic textile dyes has increased. The effects of these chemicals or their metabolites on our skin and our immune system have not been well studied. However, skin irritants have been reported to break the dermal barrier to start a chain of reactions that dysregulate the immune system. In the last decades, the incidence of atopic diseases and cancer has been increasing. There is an urgent need to identify the environmental triggers that fuel these conditions. This study aimed to investigate the effects of some of the common disperse textile dyes on the viability, and mitochondrial function of cultures of mouse keratinocytes (MPEK-BL6 line) and intestinal porcine epithelial cells (IPEC-J2 cells). The cells were cultured with Disperse (D) dyes Red 11, Orange 37, Blue 1, Blue 124, Blue 291, Blue 79.1, and Brown 1 as well as Quinone and Tartrazine, and PPD as control. At concentrations representative of human exposure from 30 min to 3 days. Cell viability, Oxygen Consumption Rate (OCR) and Extracellular Acidification Rate (ECAR) were quantified. Disperse Blue 1, Blue 124, and Brown 1 impaired cell viability and mitochondrial function as early as 3 h after exposure with IPEC-J2 and MPEK-BL6 cells. However, D. Blue 79.1 and Blue 291 did not have those effects. These data suggest that common disperse textile dyes can influence cell viability and mitochondrial function. This effect could be related to their chemical structure and physicochemical properties, such as size and polarity giving them differences in membrane permeability.

## 1 Introduction

Disperse anthraquinone and azo-benzene dyes are used to dye synthetic fabrics. They are the most abundant and fastest-growing (1). Generally, disperse dyes are derivatives of azo, anthraquinone, nitro, and quinine groups (2–4). Being hydrophobic, they do not bind to fibers and are shed from textiles, including clothing and upholstery, and they have been shown to accumulate in indoor dust particles (4–6). Environmental accumulation of dispersed azo dyes is becoming a health concern, due to its possible role as a contact sensitizer and in allergic sensitization (7, 8). We know that certain dyes can be carcinogenic, but less is known about how their chemical composition can influence their absorbance and affect the immune system (9–12). Azo dyes present in the fabric can release carcinogenic amines that can be leached out from the fabric and be absorbed due to human perspiration or sweat and transferred into the body through skin pores and then reduced during metabolism by skin bacteria, intestinal, or liver enzymes. A reduction of the microflora of the skin has also been reported (13, 14). There is an emerging need to characterize not only the dyes that pose a health issue, but also the metabolites and the maximum allowed concentrations (14).

In most cancer cell lines, the majority of acidification is due to glycolysis, a process that is used by virtually all cell types. Dividing cells rely heavily on glycolysis (15). On the other hand, glucose has traditionally been held as the primary fuel for T cell metabolic needs and T effector function. Glucose is rapidly taken up by activated T cells and is a major substrate fueling central carbon metabolism. It has also been linked to regulation of effector cytokine production by CD8+ T cells (16). Inflamed tissue consumes oxygen more rapidly by an increased oxygen consumption by infiltrating immune cells (17). Mitochondria not only provides energy (ATP), but also are required for cell viability, survival and normal cell function (17). Their signal transduction machinery regulates physiological activities such as intracellular signaling, regulates reactive oxygen species (ROS) and Ca2+ signaling, apoptosis, autophagy, and glucose-insulin regulation (17). Mitochondrial changes or damages have been associated with impairment of cellular metabolism and viability in injuries and diseases (18, 19). Alterations in mitochondrial number and fitness consequently alter T-cell fate and function (20). Damage signals released from mitochondria trigger inflammatory response pathways (21, 22).

According to Milbern et al. disperse dyes with very similar structures can lead to different colors. Minor atomic or conformational differences in their structures can also lead to differences in their solubility and toxicity (3). We hypothesize that these minor differences can probably have differential immunological effects on an organism.

This study aimed to investigate the effects of some of the common Disperse textile dyes in the viability, and mitochondrial function of cultures of two cell line models for mouse skin keratinocytes (MPEK-BL6 line) and intestinal porcine epithelial cells (IPEC-J2 cells). The cells were cultured with Disperse (D) dyes Red 11, Orange 37, Brown 1, Blue 1, Blue 124, Blue 291, Blue 79.1, as well as Quinone and Tartrazine and p-Phenylenediamine (PPD) as control, dissolved in acetone or DMSO at low and high concentrations representative of human exposure for 30 min to 3 days. PPD was used as a positive control due to its already known high toxicity in cells (23).

## 2 Materials and methods

### 2.1 Dyes and cell cultures

The following disperse (D) dyes were used in this study: D. Blue 1 (Sigma 215643-5G), D. Blue 124 (21620-5G), D. Blue 291(TRC-D495358), D. Blue 79.1 (75497-74-4), D. Brown 1 (S468061-1G), D. Orange 37 (21603-5G), D. Red 11 (S944556-50MG), Tartrazine (T0388-100G), 2-Hydroxy-1,4-naphthoquinone, Quinone (H46805-10G), p-Phenylenediamine, PPD (P6001-50G). Solubility was tested in different solvents and at different concentrations to determine the optimal solubility of all dyes and with the highest cell viability and low toxicity. Acetone and DMSO (Corning 25-950-CQC) were selected as solvents. To calculate the amount of dye needed to expose the cells to be representative of the surface area of human exposure, the dye calculations were done as follows: A regular small T-shirt weights ∼200–150 g and ∼3 g of dye is used to stain them. There are ∼4 × 10^6^ cells/in^2^ in human skin, a T-shirt has a surface of ∼767 in^2^. Our T75 flasks have a surface area of 75 cm^2^ and our 96 well plates of 3 mm^2^/well = 0.00465 in^2^ equivalent to ∼18.6 × 10^3^ skin cells. We seed our 96 well plates at 2–5 × 10^5^ c/well. Therefore, 18 μg of dye/well would be representative of the exposure with a T-shirt with a surface of 767 in^2^ with 3 g of dye and 18.6 × 10^3^ skin cells. Since our wells have 5 × 10^5^ c/well, the total amount of dye for a 96 well plate would be 483.87 μg/well. This amount would be considered the high dose in this experiment. A dilution of 1:10 = 48.387 μg/well would be considered the low dose.

Cultures of IPEC-J2 were incubated with the different synthetic textile dyes in DMEM-F12 50/50 (Corning 10-090-CV) with supplements. MPEK-BL6 cell lines were cultured in CnT-PR media (ZenBio, CELLnTEC, Durham, USA). Cells were incubated with 5% CO_2_ at 37°C either in 96 well plates or in T 75 flasks.

### 2.2 Cell viability—LUNA counts and CellTox green cytotoxicity assay

**Figure 1:**
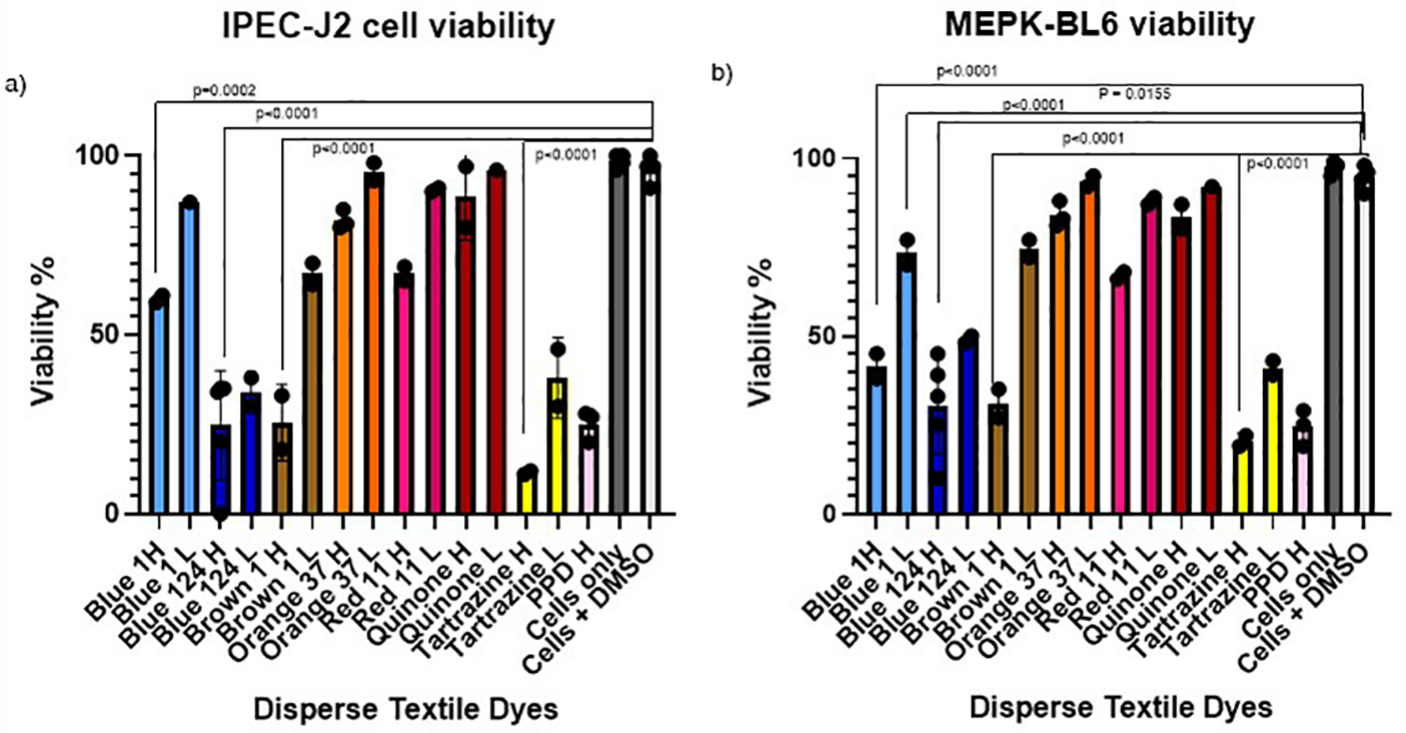
**(a,b)** IPEC-J2 and MPEK-BL6 cell viability after exposure with the different disperse textile dyes at high and low concentrations after 1 day in culture. Cell viability was significantly decreased when cultures of mouse keratinocytes (MPEK-BL6 line) and intestinal porcine epithelial cells (IPEC-J2 cells) were co-cultured with disperse dyes Brown 1, Blue 124, Blue 1 and Tartrazine after 1 day exposure in culture.

MPEK-BL6 and IPEC-J2 cells were cultured in T75 flasks at a concentration of 2 × 10^6^ cells at 80% confluency and exposed for several time points starting from 30 min to investigate acute effects on cell viability, up until 3 days to investigate the longer exposure leading to irreversible and toxic effects on the high (H) and low (L) concentrations with the different disperse dyes. Cells were observed under a Keyence digital microscope and then harvested and counted on an automated LUNA-II cell counter. Viability was measured and compared to cells alone or to cells in contact only with the solvents (Figure 1).

**Figure 2:**
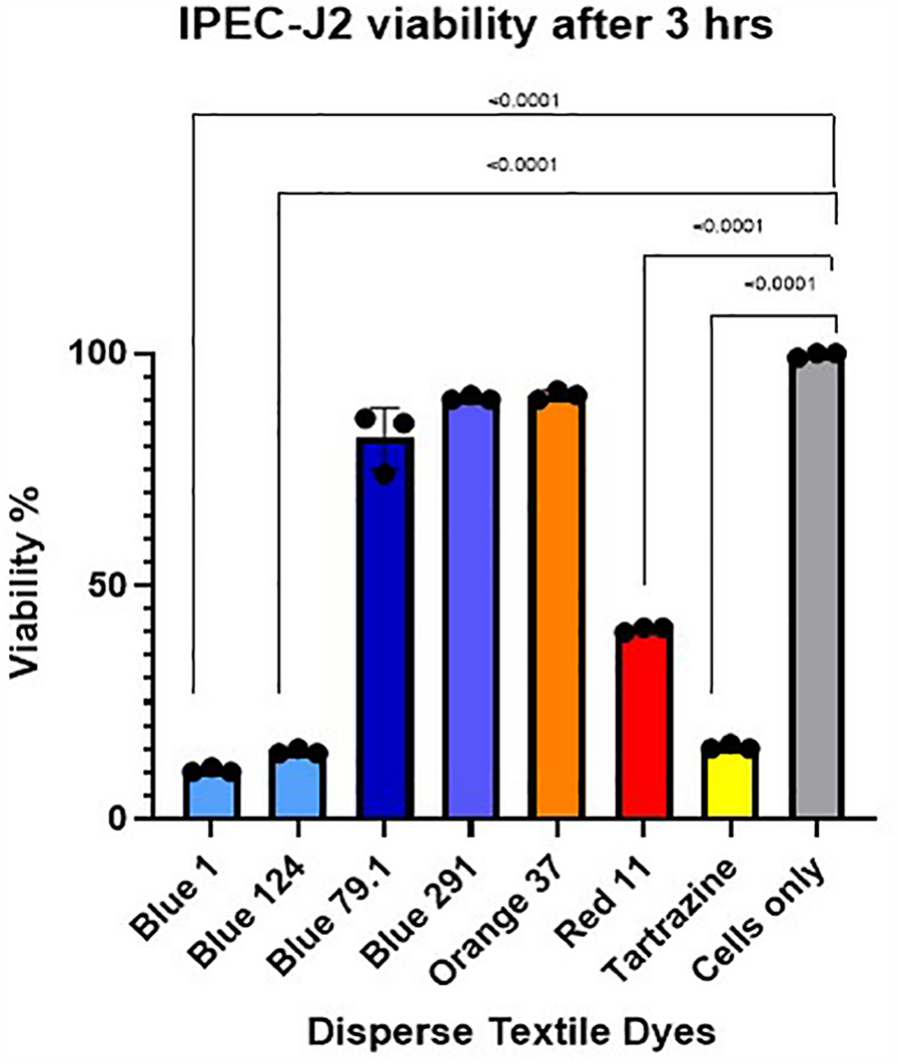
IPEC-J2 cell viability after 3 h exposure with high concentration of the different disperse textile dyes using cell Tox green cytotoxicity assay. Tartrazine, D. Red 11, D. Blue 1 (268.2760 g/mol) and D. Blue 124 (377.4190 g/mol) had significant *p* < 0.0001 cell toxicity but not D. Blue 291 (509.3170 g/mol) or D. Blue 79.1 (625.3890 g/mol), which have a greater mass and different polarities.

In order to measure changes in membrane integrity that occur as a result of cell death after disperse dyes exposure, we used CellTox Green Cytotoxicity Assay (G8742 Promega). IPEC-J2 cells were adjusted to 20,000 cells/well, plated on 50 µl of cells to a black 96 well plate (Thermo Scientific 165305) also wells with no cells were included for the background control. Cells were attached overnight and then the disperse dyes to be tested were added at the high and low concentrations for several time points starting from 30 min to 3 days. Lysis solution was added to some wells as a positive control. Staining was done according to the protocol. Cells were incubated with the reagents and then read fluorescence at 485–500 nmEx/520–530 nmEm. Cell viability from each dye was compared to cells alone or cells with the solvents (Figure 2).

### 2.3 Mitochondrial respiration

**Figure 3:**
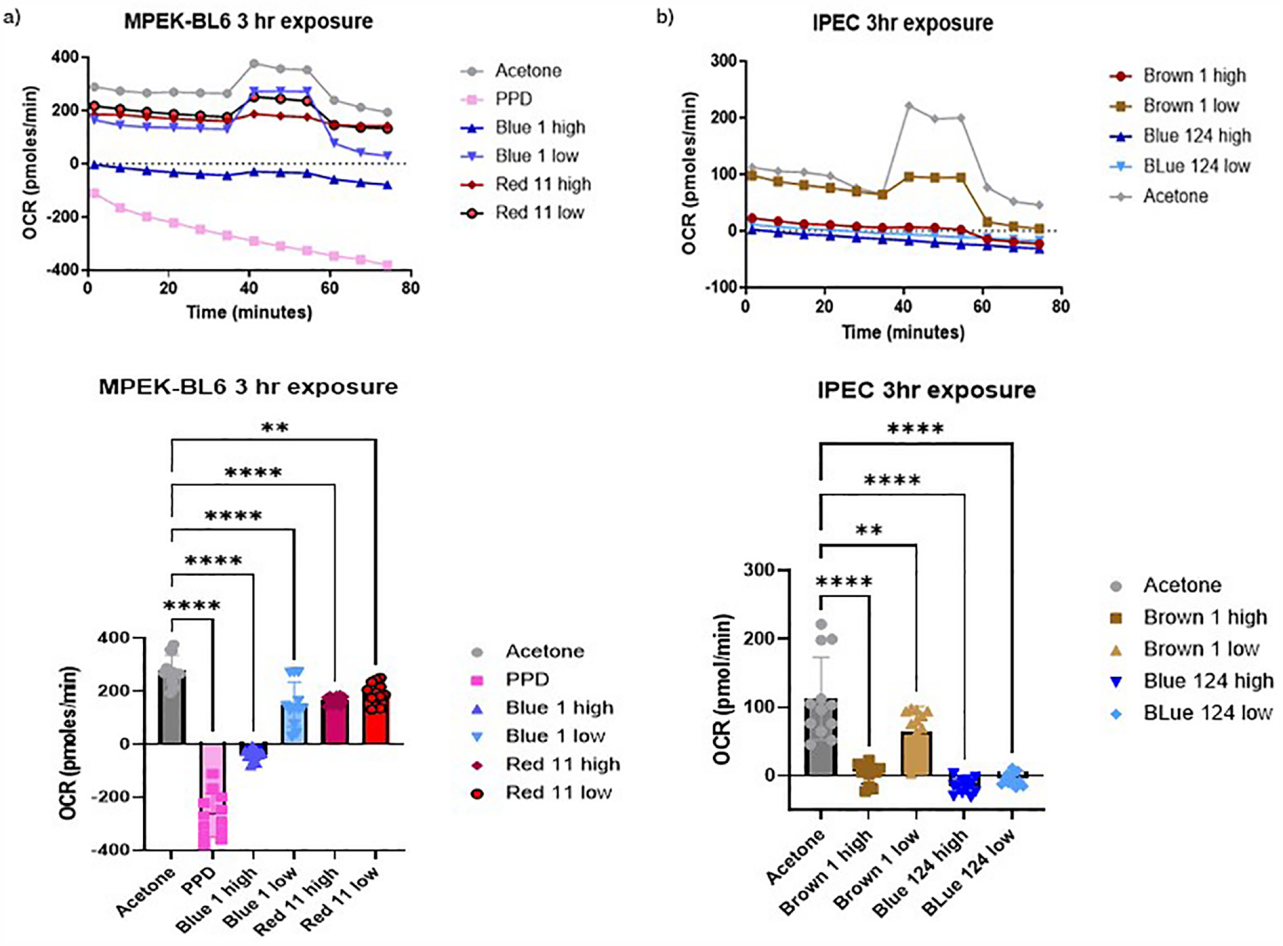
**(a,b)** Oxygen consumption rate (OCR) of IPEC-J2 and MPEK-BL6 cells was evaluated using seahorse XF cell mito stress test Kit. This figure shows MPEK-BL6 and IPEC-J2 OCRs when exposed to a high or low concentration of the disperse textile dyes for 3 h. Disperse Brown 1, Blue 124 and Blue 1 significantly *p* < 0.001 inhibited ORC mitochondria) respiration compared to the other dyes.

**Figure 4:**
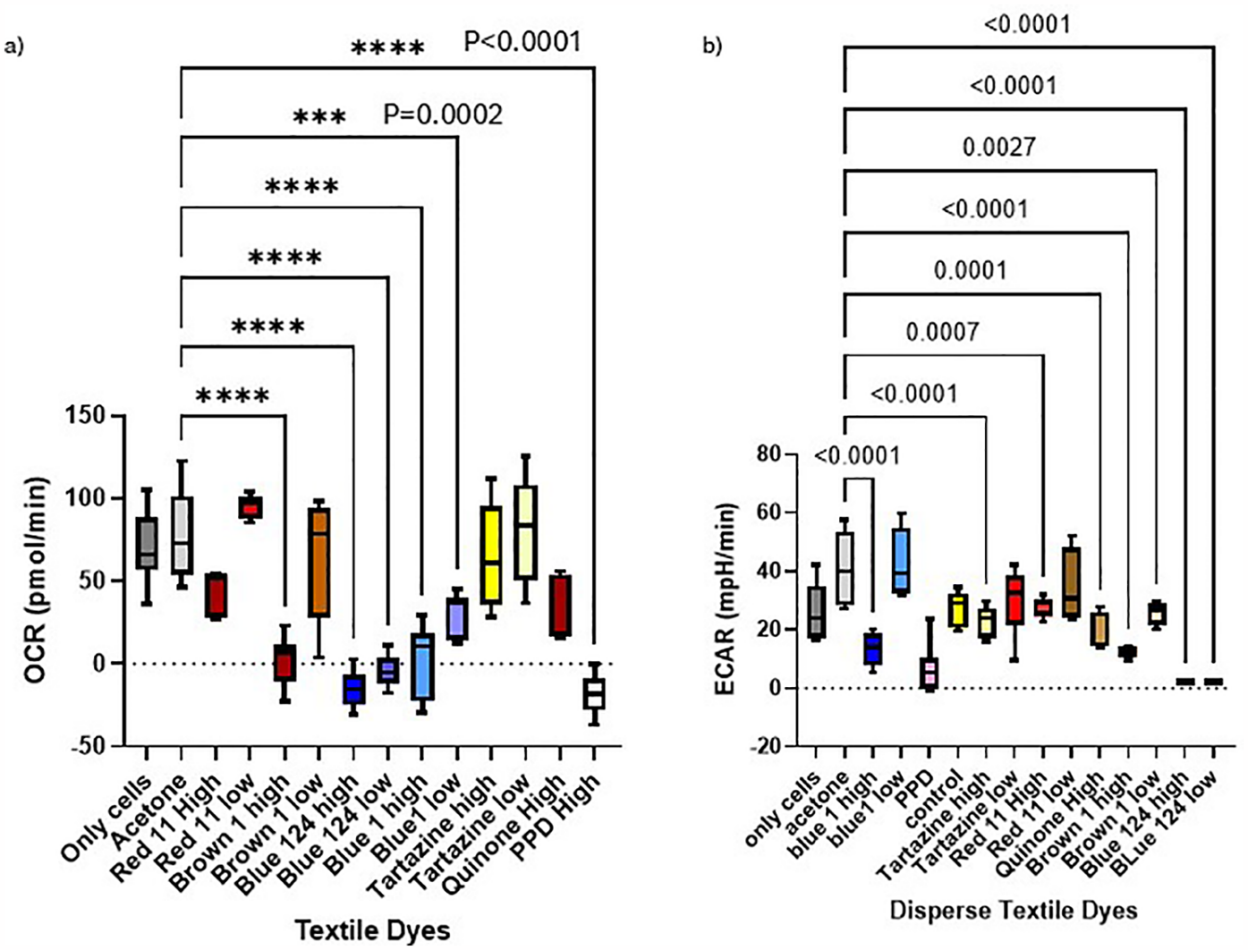
**(a,b)** Oxygen consumption rate (OCR) and ECAR of IPEC-J2 and MPEK-BL6 cells after 1 day exposure with the dyes. This figure shows MPEK-BL6 and IPEC-J2, OCRs and ECAR measurements by Seahorse XF Cell Mito Stress Test Kit when exposed to a high or low concentration of the disperse textile dyes for 1 day. Disperse Brown 1, Blue 124 and Blue 1 significantly inhibited ORC mitochondrial respiration compared to the other dyes and had a significant change in glycolysis measured by ECAR.

Agilent Seahorse XF Cell Mito Stress Tests Kits (MSIPS4W10, Millipore) were used to assess mitochondrial function. Oxygen consumption rate (OCR) and Extracellular acidification rate (ECAR) were quantified to evaluate mitochondrial respiration. IPEC-J2 and MPEK-BL6 cells were plated at 0.5 × 10^5^ cells/well with a final volume of 150 μl/well. Cells alone or in the presence of acetone were used as negative controls. Cells were incubated overnight with 5% CO_2_ at 37°C for attachment. Next day disperse dyes were added at high (H) and low (L) concentrations per well adjusted proportionally to the well volume. The cells were incubated with 5% CO_2_ at 37°C at various time points after exposure to capture the onset and progression of changes. Immediate and up to 30 min to investigate acute effects from inhibitors or uncouplers on mitochondrial respiration (data not shown), 1–3 h to observe changes related to intermediate or reversible effects (Figure 3) and 24–72 h longer-term (Figure 4), for more gradual or indirect effects from chemicals like oxidative stressors that can give irreversible and toxic effects. Calibration and test plates were prepared according to the protocol and incubated overnight in a non-CO_2_ 37°C incubator. Stocks were prepared with fresh complete ShM plus with Oligomycin, FCCP and RA according to the manufacturer's protocol. Data was analyzed by the Wave software (Agilent, USA). We assessed cellular functions and energy metabolism in response to textile dyes exposure.

### 2.4 Statistical analysis

One-way ordinary ANOVA tests with no pairing were used to compare the mean of each textile dye to the mean of a solvent control group to test the effects on mitochondrial respiration, as well as to analyze the cell viability of the different dyes compared to controls. All analyses were done with GraphPad Prism version 10.2.3 (San Diego, CA, USA). A *p*-value of <0.005 was considered significant.

## 3 Results

### 3.1 Cell viability and cytotoxicity

In order to test if disperse dyes could have an effect on the cell viability, we cultured our MPEK-BL6 and IPEC-J2 cells in T75 flasks in the presence or absence of the different dyes at high and low concentrations for several time points starting from 30 min to 3 days in order to test for toxicity progression and potential delayed effects. Results shown here are for 1 day exposure. The results in Figures 1a,b, corresponding to the findings done by an automated LUNA cell counter show that disperse dyes Brown 1, Blue 124, Blue 1 and Tartrazine had a very significant (*p* < 0.0001) and a slightly significant (*p* = 0.01) reduction of viability of MPEK-BL6 and IPEC-J2 cells when exposed to the high and low concentrations of these dyes, respectively.

We also measured the increase in fluorescence acquired after the cell membrane was compromised and a green cyanine dye was incorporated into the nucleus and stained their DNA. The increase in fluorescence is proportional to the cytotoxicity caused by exposing the cells to the different disperse dyes. The CellTox Green dye is in a DMSO solution. Cell tolerance to DMSO was tested first. As observed in Figure 2, analyzing viability by the CellTox Green Cytotoxicity Assay, D. Blue1 (268.2760 g/mol) and Blue 124 (377.4190 g/mol) had cell toxicity but not Blue 291 (509.3170 g/mol), or Blue 79.1 (625.38 g/mol) which have a greater mass and different polarities. As observed in Table 1, D. Blue 1 and Blue 124 have a smaller molecular weight and seem to be more planar in their structure. The microscopic images on Table 2 show that Disperse textile dyes Blue 1, Blue 124 and Brown 1 reduced the viability of MPEK-BL6 and IPEC-J2 cells, comparable to the known toxic PPD, used as a positive control for cell viability. Acetone and DMSO were used as the dye solvents and had no effect on cell viability.

**Table 1:** Chemical formula, molecular weight and chemical structure of the significant disperse dyes.

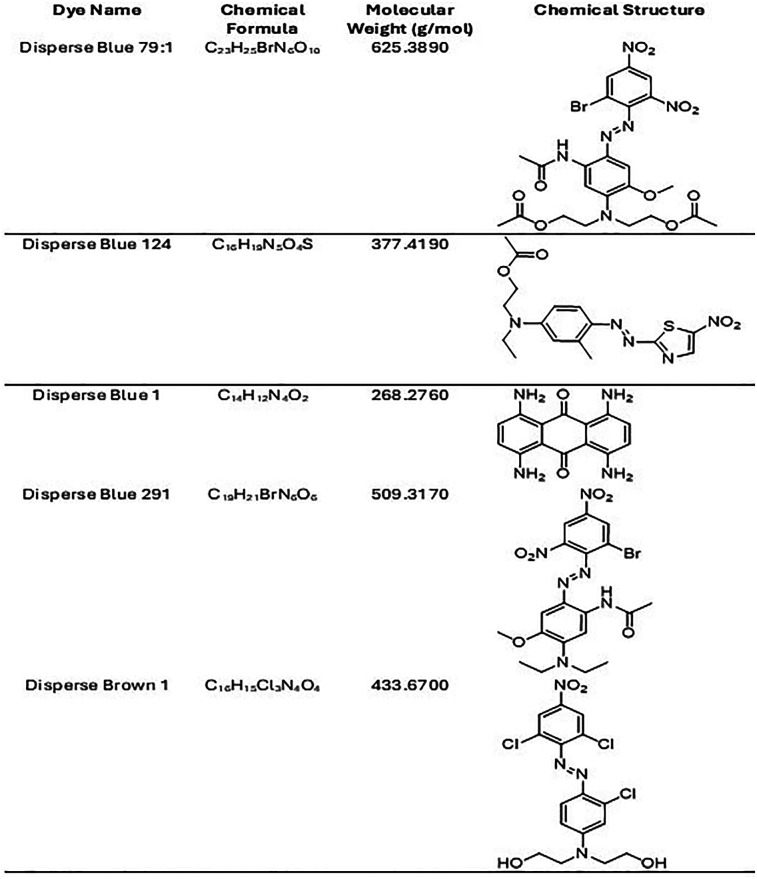

**Table 2:** Microscopic images of cultures of mouse keratinocytes (MPEK-BL6 line) in the presence or absence of the different dyes at high and low concentrations for 1 day. Microscopic cell viability analysis corresponds to the viability findings done by automated cell counter and by cytotoxicity. Disperse textile dyes Blue 1, Blue 14 and Brown 1 reduced the viability of MPEK-BL6 and IPEC-J2 cells (data not shown), comparable to the known toxic PPD, used as a positive control for cell viability. Acetone and DMSO were used as dye solvents and had no effect on cell viability.

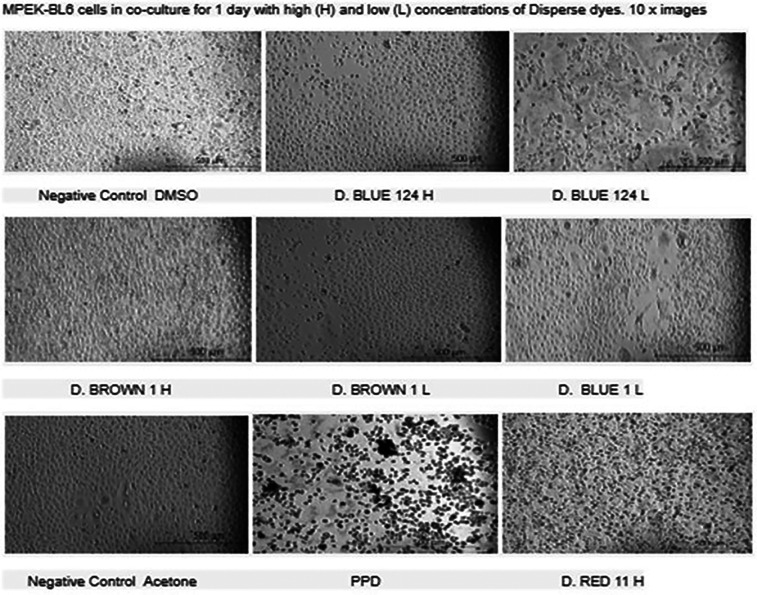

### 3.2 Mitochondrial respiration

The Seahorse Analyzer continuously measures oxygen concentration and proton flux in the cell supernatant over time. These measurements are converted into OCR and ECAR values allowing a direct quantification of mitochondrial respiration and glycolysis, respectively. In this study we investigated the basal mitochondrial function and mitochondrial stress of our MPEK-BL6 and IPEC-J2 cells once pre-exposed to the different dyes. Changes in oxygen concentration (OCR) and pH (ECAR) are continuously reported. As observed in Figures 3a,b D. Blue 1 at a high concentration, had the maximum OCR in MPEK-BL6 cells after a 3 h pre-exposure with the dyes. An indicator of failed mitochondrial respiration. D. Blue 124 had an effect in OCR even at the low concentration in IPEC-J2 cells also after 3 h exposure. D. Brown 1 had that effect at high concentration. In Figure 4a we observe the OCR rates of all the dyes tested in this article. We can observe that in IPEC-J2 cells that were in contact with the dyes for 1 day, the blue dyes D. Blue 1, and D. Blue 124 had a very significant (*p* < 0.0001 or *p* = 0.0002) effect on OCR even with the low concentrations, comparable to the very toxic PPD compound. And in Figure 4b we observe that the ECAR, a measurement of glycolysis, was also altered when IPEC-J2 cells were in contact with D. Blue 1 at high concentration or D. Blue 124 at high or low concentrations. Higher concentrations of chemicals generally lead to more immediate effects on mitochondrial respiration. Lower concentrations might require a longer exposure time to observe significant changes.

Inhibitors of mitochondrial complexes can show observable effects on mitochondrial respiration almost immediately (minutes to hours). Uncouplers can lead to rapid changes in respiration as they dissipate the proton gradient, resulting in increased oxygen consumption. Chemicals inducing oxidative stress might take longer to show their effects as they first cause damage to mitochondria, which would then affect respiration over time.

## 4 Discussion

We have shown that exposure of primary keratinocytes from mice and intestinal porcine epithelial cells to certain disperse textile dyes reduces viability and causes accelerated metabolism, manifested by a rapid mitochondrial oxygen consumption and glycolysis. In organisms this could be indicative of inflammation, as tissues with ongoing inflammation are characterized by shifts towards high rates of metabolism (16). The experiments described in this study provided the basis for future studies to investigate how exposure to specific disperse dyes can affect resident cells. Our preliminary results (data not shown) have suggested that cytokine IL-16 (that functions as a chemoattractant, and modulator of T cell activation), and chemokines such as CCL20 (that attracts lymphocytes towards epithelial cells), have been upregulated in Blue 1 exposed cells using nanostring technology.

In future studies we will characterize the mechanisms involved in the impairment of cell viability and mitochondrial function by quantifying oxidative stress markers such as, 8-hydroxy-2′-deoxyguanosine (8-OHdG), malondialdehyde (MDA) and 4-hydroxynonenal (HNE, 26).

This study is relevant since textile dyes can shed from the fabrics and have been found in indoor dust up to 6.106 μg dye/g of house dust (6). EPA estimates that children ingest, on average, 50 mg of house dust per day (US Environmental Protection Agency, 2011). This means crawling infants ingest ∼3 g of textile dyes/day. We based our exposure for dye concentrations in 3 g of dye, making this study representative of daily ingestion by infants and therefore biologically relevant.

Future studies will characterize these textile dyes by mass spectrometry as well as their metabolites before and after their interaction with skin and immune cells. We will use the historic Max Weaver Dye Library, a dye collection of 100,000 dyes (24), to determine the structural relationships of similar dyes, using machine learning, and their effects on the immune system.

We hypothesize that the greater mass and probably the stereo conformation of the structure of D. Blue 79.1 and 291 does not allow them to permeate skin or cell membranes. Future studies will describe the mechanism and investigate if the chemical and physical properties of the dyes (e.g., certain groups of azo-benzene textile dyes with similar functional groups or polarities) and their metabolites can, upon skin exposure, trigger local inflammation of the neighboring cells and activate skin immune cells.

Skin is the largest organ in our bodies, comprising up to 22 sq ft (2 sq m) in surface area (25). We wear clothes most of the time and therefore we are in continuous contact with textile dyes. However, leaching-out of the textile dyes can happen due to factors like the washing cycles which can affect the textile dye fastness by altering the dye's stability through factors like temperature, detergent type, agitation, and washing duration (6). The azo dyes subject of this study are used for dyeing polyester and they do not fast with strong bonds to the textile. Overdahl et al. found that in polyester clothing even after several washing cycles there were 21 azo dyes detected at concentrations up to 9,230 μg dye/g shirt, leaving close to half of the amount used for staining remaining in the fabric (6).

In addition, sweat and perspiration can affect textile dye absorption through the skin by increasing the likelihood of dye leaching, as sweat contains moisture, salts, urea, and various organic compounds. Its pH level (4.5 to 7.5) can influence the solubility and movement of dye molecules from fabric to skin (25). Sweat could also act as a solvent, allowing dyes to dissolve more easily from the textile, leading to color transfer through skin pores. Certain azo dyes are more likely to release into sweat than others. The amount of time a person sweats and the intensity of the sweating will influence how much dye leaches out. The chemical structure of the dye will determine how easily it can break down or migrate out of the fabric. Only a small portion of the dye that leaches from the fabric may be absorbed by the skin. The absorption rate depends on factors like skin permeability, the molecular size of the dye, and the length of contact. Dyes with larger molecular sizes could be less likely to be absorbed through the skin. Nevertheless, the continuous and repeated exposure of textile dyes can become significant. For a molecule to cross the skin barrier, it must have a pH between 4.6 and 5.5, be a lipophilic compound (log Po/w > 1) of low molecular weight, below 500–700 Daltons (25). The dispersed dyes here investigated have log P from 2.5 to 5, and molecular weights from 268 to 625 Daltons (25). Disperse Blue 1 (268.2760 g/mol) and Disperse Blue 124 (377.4190 g/mol) had cell toxicity but not Disperse Blue 291 (509.3170 g/mol), or Disperse Blue 79.1 (625.38 g/mol) which have a greater mass and different polarities.

Although we have not performed any Mass spectrometry (MS) or Machine learning studies (ML), we know that all of these dyes are hydrophobic (3). The cell toxicity and skin absorption of these dyes could be influenced by their chemical structure, molecular size, hydrophobicity, and functional groups. Disperse Brown 1 may be toxic due to the potential for reduction of the azo group and the formation of harmful aromatic amines, it has additional functional groups that could increase the potential for cellular toxicity and oxidative damage (26). Although its low water solubility limits its ability to penetrate intact skin, prolonged exposure, particularly in individuals with sensitive skin, could lead to local irritation and some degree of absorption (9). Disperse dyes are typically hydrophobic, and they tend to accumulate in tissues such as the skin and fatty organs rather than being easily excreted by the body (4). This property could lead to chronic exposure risks if the dye is absorbed over time. Disperse Blue 1 is more likely to penetrate the skin than larger molecules. Disperse Blue 79.1 and Blue 291, are predicted to have limited skin penetration due to their larger molecular structure. They are typically stable, with lower likelihoods of forming toxic metabolites. However, it can still induce cellular oxidative stress in high concentrations or with prolonged exposure.

The findings in other studies align with our current findings. Many studies corroborate the effect of dyes like D. Blue 124 in causing atopic dermatitis in humans or a dysregulation of the activities of antioxidant enzymes (27–30). In the future we are planning to use the pig as an animal model for our dyes studies. Additional studies must be conducted to clarify the cause and the mechanisms involved, in order to select and design dyes that are not harmful to humans, animals and the environment.

## Data Availability

The raw data supporting the conclusions of this article will be made available by the authors, without undue reservation.
